# Research on the relationship between social support and academic self-efficacy among college students: a multivariate empirical analysis

**DOI:** 10.3389/fpubh.2025.1507075

**Published:** 2025-02-04

**Authors:** Chunmei Chen, Yujie Zhu, Fanghao Xiao

**Affiliations:** ^1^Teachers College, Jimei University, Xiamen, Fujian, China; ^2^Faculty of Education, The University of Hong Kong, Hong Kong, China; ^3^Marxist College, Xiamen Institute of Technology, Xiamen, Fujian, China

**Keywords:** social support, academic self-efficacy, sense of security, family cultural capital, mediating effect, moderating effect

## Abstract

**Background:**

The critical role of social support in college students’ academic development cannot be ignored. This study aims to analyze the interrelationships and potential mechanisms between social support, sense of security, family cultural capital and academic self-efficacy.

**Methods:**

A multivariate moderated mediation model was constructed by surveying 1,119 college students using the Social Support Scale, the Sense of Security Scale, the Academic Self-efficacy Scale, and the Family Cultural Capital Scale.

**Results:**

(1) social support significantly increases sense of security; (2) sense of security significantly enhances academic self-efficacy; (3) social support directly contributes to academic self-efficacy; and (4) sense of security partially mediates the relationship between social support and academic self-efficacy; (5) family cultural capital plays a moderating role in the effects of social support on academic self-efficacy, especially in the first half of the direct and indirect effects.

**Conclusion:**

These findings could provide an important theoretical basis and practical guidance for further understanding and enhancing academic self-efficacy among college students.

## Introduction

Self-efficacy refers to an individual’s expectation of his or her ability to operate a behavior in a given context, including outcome expectations (an individual’s prediction that a behavior will lead to a particular outcome) and efficacy expectations (an individual’s expectation that he or she will be able to carry out a behavior successfully in order to produce a specific outcome) (1). It affects an individual’s behavioral choices, motivational efforts, cognitive and affective processes (2). Specifically, self-efficacy affects people’s choice of activity and persistence in that activity, as well as their attitudes toward difficulties and their patterns of thinking and emotional responses. Academic self-efficacy is a manifestation of an individual’s academic self-efficacy, which mainly refers to an individual’s judgment and confidence in his or her ability to successfully complete academic tasks (3, 4). Schunk views it as the confidence and expectation of a student’s ability to accomplish a set goal during the learning process (5). Studies have confirmed that academic self-efficacy is closely related to college students’ learning burnout (3, 6), academic achievement motivation (7, 8), and academic adjustment (9). College students lacking academic self-efficacy are more likely to fall into self-doubt and negative thinking, leading to confusion and lack of motivation in the learning process. They are prone to anxiety, frustration, and fear and give up when facing academic challenges, making it difficult for them to achieve academic success. Academic self-efficacy is of great significance to the academic development of college students and deserves more attention from scholars. Several studies have also pointed out that social support has a positive predictive effect on academic self-efficacy (10–13). In addition, academic self-efficacy may also be related to sense of security (14, 15) and family cultural capital (16). From the social cognitive theory perspective, social factors (e.g., social support and cultural background) and emotions may impact an individual’s academic self-efficacy (17). Currently, more studies directly explore the two-way relationship between social support, sense of security, family cultural capital, and academic self-efficacy. However, few studies simultaneously explore the relationship between these variables and the potential mechanisms of influence. The present study hopes to make a breakthrough in this area.

### The relationship between social support and sense of security

The social support theory emphasizes that various kind of supports obtained from social networks in social life can alleviate the negative effects of stress and thus improve individual’s overall health level and quality of life. Caplan, Cassel and Cobb play an important role in the development of the theory (18–20). Social support is the sum of various kinds of help and support that an individual receives from the surrounding social network in the process of interacting with others, including emotional support (e.g., providing comfort and encouragement) and instrumental support (e.g., providing material support or practical behavioral help) (21). In addition, social support also includes informational support (e.g., providing knowledge and information) and evaluative support (e.g., providing feedback on achievements and problems) (22). Social support facilitates individuals to better cope with stressful events (23, 24). The sense of security is an individual’s preconceived notion of possible dangers or risks to his or her body or psyche from the surrounding environment, as well as the individual’s feelings (power or powerlessness) in coping with the dispositions, which are mainly manifested as a sense of certainty and a sense of controllability (25–27). Owning a higher level of social support is an important factor in maintaining and increasing an individual’s sense of security. Students feel more secure when they feel support from organizations, friends, and family (28). Social support is significantly and positively related to sense of security (29–31). In summary, hypothesis H1 is proposed:

*H*1: There is a significant effect of social support on sense of security.

### The relationship between sense of security and academic self-efficacy

Psychology has done much security research. Freud, the psychoanalytic school, firstly focuses on studying the sense of security. He believes that the sense of security arises in an individual’s early childhood, and whether specific desires and needs can be satisfied in the process of growth of an individual affects the development of the sense of security to a great extent (32). However, it is humanistic psychologist Maslow who formalized the sense of security as a concept. He categorizes human needs into five levels: security is the second. Sense of security is viewed as a feeling of confidence, safety from fear and anxiety, and in particular, the ability to meet a variety of an individual’s present and future needs. Lack of security can have a range of adverse effects on an individual’s physical and mental health (33). Research on nursing students has found that a sense of security helps to boost their confidence and better cope with difficulties and challenges in their academic life as well as in their future work (15). Students with a higher sense of security are more willing to demonstrate their abilities relative to others in the learning process. They can participate more actively in class and are more motivated to learn, reporting higher levels of academic self-efficacy (34). Sense of security are positively related to academic self-efficacy (35, 36). In summary, hypothesis H2 is proposed:

*H*2: There is a significant effect of sense of security on academic self-efficacy.

### Relationship between social support and academic self-efficacy

Teachers, parents, and peers are important sources of social support for students. Teachers provide social support to students by considering students’ perspectives, promoting open communication, and providing students with choices and feedback (37). The support given by parents enables students to hold a higher sense of value and their motivation to complete academic tasks is more significant (38). In addition, social support from peers is a more significant predictor of increased academic self-efficacy (39). A study of 1,048 college students from Spain has found that when students feel social support, it can mobilize their more profound learning methods, which can help improve their perceived professional ability and academic self-efficacy (40). In addition, social support can provide students with emotional value (e.g., warmth, comfort) and buffer the effects of school stress. The higher the level of students’ sense of social support, the more enthusiastic they are about subject learning (41). Social support is a significant predictor of academic self-efficacy (42–45). In summary, hypothesis H3 is proposed:

*H*3: There is a significant effect of social support on academic self-efficacy.

### Mediating effects of sense of security

A sense of security is the feeling of certainty and positivity associated with the experience of trustworthiness, reliability, and serenity resulting from a person’s ability to solve problems positively (46). Students with a low sense of social support lack positive peer interactions and social interactions. They are more vulnerable to risk-taking for certain stressful life events and tend to have lower levels of adaptation to various social environments, leading to their lower sense of security (47). Social support positively predicts sense of security (48–50). Some researchers put forward that risk factors during the COVID-19 epidemic lead to an increase in students’ real-world insecurity. Especially in academic life, the blockage of various real-life learning resources hinders students’ desire to learn. Uneasiness makes it difficult for students to concentrate on obtaining a corresponding sense of achievement and satisfaction in learning, reducing their academic self-efficacy (51). Sense of security positively predicts academic self-efficacy (52, 53). In summary, hypothesis H4 is proposed:

*H*4: Sense of security mediates the relationship between social support and academic self-efficacy.

### Moderating mediating effect of family cultural capital

Cultural capital is an important sociological theory proposed by Bourdieu, a famous French sociologist. The theory focuses on how cultural factors affect the reproduction of social classes and the role of culture as a kind of capital in social life. The term “cultural capital” represents individuals’ skills, knowledge, education, and interests that give them a place in society (54). According to Bourdieu and Passeron, cultural capital is an asset related to cultural activities, which can be categorized into concrete, institutional and objective forms (55). Family cultural capital is the manifestation of cultural capital in the specific field of the family. It demonstrates the family’s accumulation and transmission of cultural capital (56). Some scholars classify family cultural capital into concrete family cultural capital (e.g., family cultural atmosphere, parents’ reading habits and educational expectations, etc.), institutionalized family cultural capital (e.g., parents’ level of education), and objectified family cultural capital (e.g., good books and learning tools purchased by the family) (57, 58). Family cultural capital, by giving access to rich cultural resources, can help create a safe environment for students, increase their sense of security and promote their extracurricular engagement. Students with higher family cultural capital are more likely to succeed academically and in their future careers (59). Family cultural capital is positively correlated with sense of security (60, 61). Additionally, students with lower family cultural capital have a more prominent likelihood of academic mismatch, which is detrimental to their successful transition to college. They may take longer to earn a degree and have a lower probability of graduating (62). Students whose families have higher cultural capital tend to have better academic performance and more academically positive, positive emotional experiences when they are exposed to cultural resources. They may take longer to earn a degree and have a lower probability of graduating. Students whose families have higher cultural capital tend to have better academic performance and more academically positive, emotionally charged experiences when exposed to cultural resources. They have higher academic self-efficacy (63, 64). There is also a positive correlation between family cultural capital and academic self-efficacy (65, 66). In summary, hypothesis H5 is proposed:

*H*5: Family cultural capital moderates the mediating effect of social support and academic self-efficacy.

**Figure 1:**
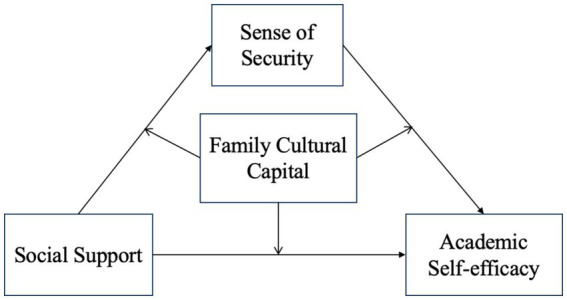
Theoretical model.

This study constructs a moderated mediation model to explore the mechanism of social support on academic self-efficacy in a group of college students, with a view to providing new ideas for promoting college students’ learning. The theoretical model is shown in Figure 1.

## Methods

### Data sources and sample characteristics

This study was conducted according to the guidelines of the Declaration of Helsinki and approved by the Ethics Committee of Jimei University. Informed consent was obtained from all participants involved in this study. The sample survey used the convenience sampling method, covering a total of 1,300 full-time college students from different types of domestic colleges and universities such as Jimei University, Xiamen Institute of Technology, and Wuxi Taihu College. Before filling out the questionnaire, the participants were informed of the purpose of the study, its procedures, its potential risks and their right to volunteer for the study. They can withdraw at any time during the questionnaire completion process and put forward any questions if needed. By screening invalid samples (reverse questions, trap questions), 1,191 valid samples were obtained, and the effective rate of the questionnaire was about 91.6%. The gender ratio was close to balanced, with 49.24% males and 50.76% females; the age concentration was mainly between 18 and 21 years old (94.28%), which is in line with the common age distribution of the undergraduate student population. In terms of majors, 52.46% of the students were in science and engineering and 47.54% were in arts and sciences, showing a good representation of disciplines for further research. The basic characteristics of the sample are shown in Table 1.

**Table 1:** Descriptive statistics of the sample.

Name	Option	Frequency	Percentage (%)
Gender	Male	551	49.24
	Female	568	50.76
Age Group	Under 18	34	3.04
	18–21 years old	1,055	94.28
	21–24 years old	26	2.32
	24–27 years old	4	0.36
Major	Science and Engineering	587	52.46
	Arts	532	47.54
Total		1,119	100.0

### Research instruments

In this study, the KMO values for the social support, sense of security, academic self-efficacy, and family cultural capital scales were 0.912, 0.934, 0.895 and 0.891, respectively. It is mentioned that the KMO values between 0.8 and 0.9 are considered great, and above 0.9 are deemed superb (67). All of these values are significantly above 0.8, indicating that the data are suitable for factor analysis. Additionally, the Cronbach’s *α* coefficients for these scales were 0.897, 0.892, 0.867, and 0.912, all exceeding 0.8. According to the standard for Cronbach’s α, a value above 0.8 indicates high reliability (68). This indicates that the scales exhibit good internal consistency. Thus, the reliability and validity of all scales meet the necessary standards for statistical analysis, demonstrating strong operational validity and reliability.

### Social support scale

The Social Support Scale for Adolescents developed by Ye et al. (69) was used in this study. The scale consists of three sub-dimensions. Subjective support items such as “Most of my classmates care about me”; Objective support items such as “I can get financial support from my family, friends and relatives when I need it” and utilization support items such as “When faced with a dilemma, I will take the initiative to ask for help from others” etc. The scale consists of 17 entries and is rated on a five-point scale. The KMO value of the scale was 0.912, and the study data were well suited for extracting information; the Cronbach *α* coefficient of the scale was 0.897, and the scale had good consistency with valid measurements. All question items were summed and averaged to obtain the variable social support, which was used to indicate the level of social support. Higher scores indicated higher levels of social support.

### Sense of security scale

The scale was developed by Cong and An in 2003 (70). The scale consists of 16 items on a five-point scale. Items such as “I never dare to volunteer my opinion,” “I feel that life is always full of uncertainty and unpredictability,” “I am always worried that my life will become a mess” etc. The KMO value of the scale was 0.934, and the study data were well suited for extracting information; the Cronbach *α* coefficient of the scale was 0.892, and the scale had good consistency with valid measurements. After reversing the scoring of the reverse questions in it, all the items were summed and averaged to obtain the variable sense of security, which was used to indicate the degree of sense of security. Higher scores indicated a higher sense of security.

### Academic self-efficacy scale

The Academic Self-efficacy Questionnaire for College Students developed by Liang in 2002 (71) was used in this study. The scale consists of 22 items and is rated on a five-point scale. Questions such as “I believe I have the ability to do well in my studies,” “Compared with other students in my class, I am relatively strong in my studies,” “I often fail to accurately summarize the main meaning of what I read “etc. The KMO value of the scale was 0.895, and the study data were well suited for extracting information; the Cronbach *α* coefficient of the scale was 0.867, and the scale had good consistency with valid measurements. After reversing the scoring of all questions in it, all the items were summed and averaged to obtain the variable academic self-efficacy, which was used to indicate the degree of academic self-efficacy. Higher scores indicated higher levels of academic self-efficacy.

### Family cultural capital scale

The scale was developed by Kuang in 2021 (58). The scale consists of 13 items on a five-point scale. Items such as “Your family atmosphere is harmonious and loving,” “Your mother often reads and studies at home,” “Your parents pay a lot of attention to cultivating your hobbies” etc. The KMO value of the scale was 0.891, and the study data were well suited for extracting information; the Cronbach *α* coefficient of the scale was 0.912, and the scale had good consistency with valid measurements. After reversing the scoring of the reverse questions in it, all the items were summed and averaged to obtain the variable family cultural capital scale, which was used to indicate the degree of family cultural capital scale. Higher scores indicated higher levels of family cultural capital scale.

### Research design and data processing

In this study, we adopted a quantitative research design. Descriptive statistics and Pearson correlation analysis were executed in SPSS 26.0. Concretely, Descriptive statistics were analyzed to understand the basic characteristics of the sample and Pearson correlation analysis was used to explore the linear relationship between the main variables. Moreover, we used Model 59 from the PROCESS plug-in provided by Hayes in 2017 (72), which is a mediation with moderation model for analyzing complex relationships between independent, dependent, and moderating variables. Specifically, Model 59 examined the moderating effects of three paths: the effect of the independent variable on the mediating variable, the direct effect of the independent variable on the dependent variable, and the indirect effect of the mediating variable on the dependent variable. The core of the moderating effects is to explore how the moderating variable affects the strength and direction of these paths, which in turn reveals how the mediating and direct effects change under different conditions (e.g., different levels of the moderating variable). Mediation models of moderation (e.g., Model 59) allow researchers to explore both mediating and moderating effects, which enables us to analyze how individuals are affected by independent variables through mediating variables (e.g., attitudes, behaviors, etc.) under different conditions of moderating variables. Specifically, Model 59 can help analyze the following three aspects of moderation:

(1) The moderating effect of the independent variable on the mediating variable: i.e., whether the moderating variable affects how the independent variable acts through the mediating variable.
(2) The direct moderating effect of the independent variable on the dependent variable: i.e., whether the moderating variable changes the direct effect of the independent variable on the dependent variable.
(3) Indirect moderating effect of the mediating variable on the dependent variable: i.e., whether the moderating variable affects the indirect path of the mediating variable on the dependent variable.

This model is suitable for analyzing how causality and effect mechanisms change under different conditions. The significance and strength of the mediating and direct effects in moderation can be effectively assessed by testing the significance of these pathways using the bias-corrected percentile Bootstrap method. The significance of the moderated effects was tested by the bias-corrected percentile Bootstrap method with 99% confidence intervals, which were considered statistically significant only if the confidence interval did not contain zero (73). All variables were standardized to ensure the accuracy of the moderated effects analysis.

## Research results

### Common method bias test

The self-report method was used to collect data for this study. In order to assess potential common method bias, a Harman single-factor test was conducted in this study (74). The test results showed that the total number of factors with an eigenroot exceeding 1 was 13, while the first common factor explained 27.263% of the total variance, which did not exceed the critical value of 40%. Therefore, there was no significant common method bias in the data of this study.

### Descriptive statistics and correlation analysis of the variables

Table 2 showed the results of the correlation analysis between the four variables: social support, sense of security, academic self-efficacy, and family cultural capital. As can be seen from the table, positive correlations were shown between all variables, with academic self-efficacy demonstrating a strong correlation with social support (*r* = 0.531, *p* < 0.01) and family cultural capital (*r* = 0.499, *p* < 0.01). The correlation coefficient between social support and family cultural capital was 0.480 (*p* < 0.01), also showing a significant positive relationship. Sense of security, although relatively low in correlation with other variables, was still statistically significant, especially with academic self-efficacy (*r* = 0.203, *p* < 0.01). These results suggested that social support and family cultural capital have a positive impact on enhancing an individual’s sense of security and academic self-efficacy.

**Table 2:** Correlation analysis between variables.

	Mean	Standard deviation	Social support	Sense of security	Academic self-efficacy	Family cultural capital
Social support	3.438	0.670	1			
Sense of security	3.134	0.706	0.159**	1		
Academic self-efficacy	3.246	0.547	0.531**	0.203**	1	
Family cultural capital	3.024	0.627	0.480**	0.114**	0.499**	1

*indicates *p* < 0.05, **indicates *p* < 0.01, ***indicates *p* < 0.001.

### Mediating role test

**Figure 2:**
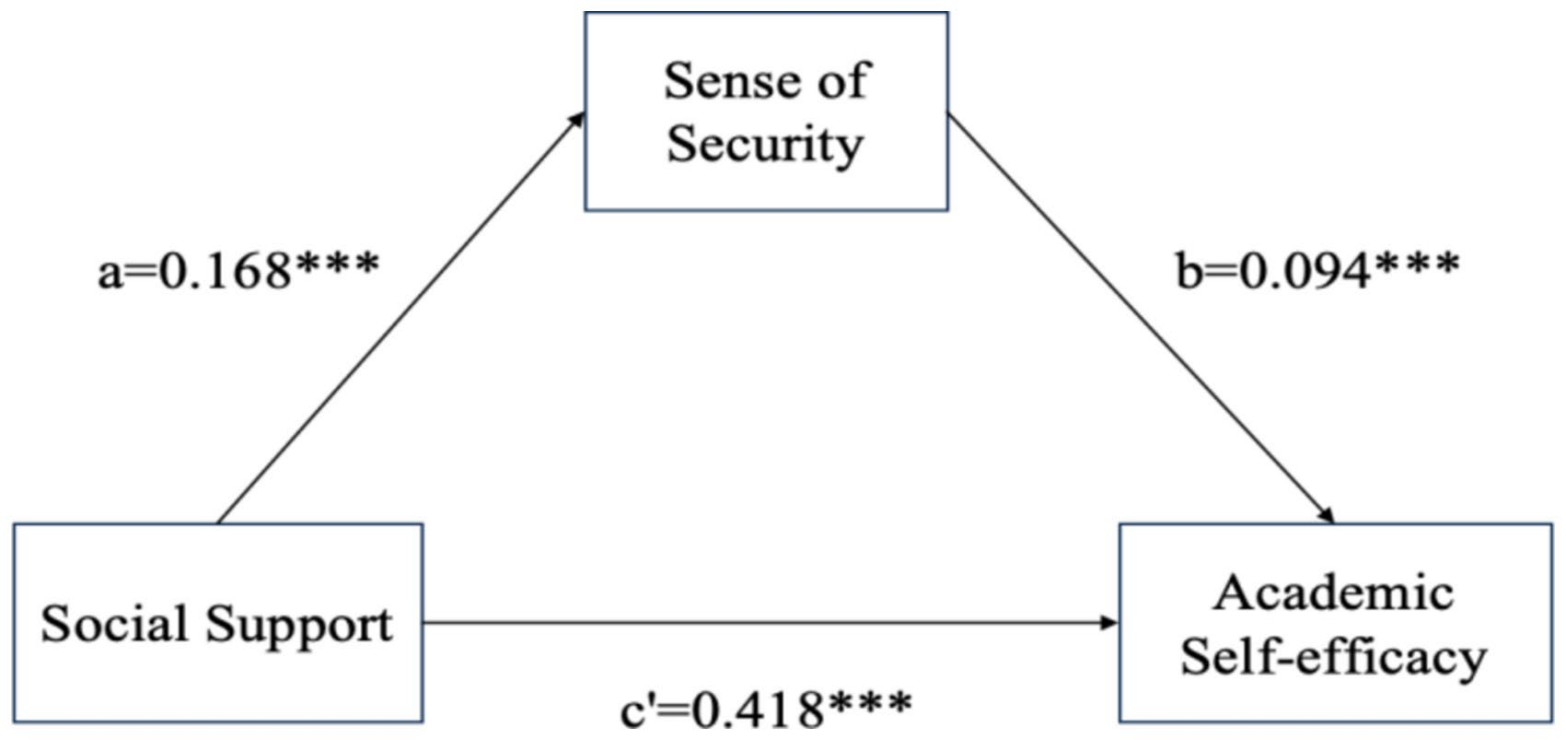
Mediation model: effect values.

Model4 (Model4 is a simple mediation model) in the SPSS macro developed by Hayes in 2017 (72) was used to test the mediating effect of a sense of security in the relationship between social support and academic self-efficacy. The results were shown in Tables 3, 4 and Figure 2. In the first regression equation analysis, social support had a significant positive effect on academic self-efficacy (*β* = 0.434, *p* < 0.01), explaining 28.1% of the variance in academic self-efficacy (R^2^ = 0.282, Adjusted R^2^ = 0.281), with a highly significant model (*F* = 438.825, *p* < 0.000). The analysis of the second regression equation showed that social support equally significantly influenced security (*β* = 0.168, *p* < 0.01), despite a lower explanatory power (R^2^ = 0.025, adjusted R^2^ = 0.024) and statistical significance (*F* = 28.970, *p* < 0.000). The analysis of the third regression equation revealed that the direct effect of social support on academic self-efficacy decreased after accounting for the potential mediating role of a sense of security (*β* = 0.418, *p* < 0.01), while the significant effect of a sense of security (*β* = 0.094, *p* < 0.01) implied its mediating role, and the explanatory power of this regression equation was higher (R^2^ = 0.297, adjusted R^2^ = 0.295), with strong overall significance (*F* = 235.183, *p* < 0.000).

**Table 3:** Mediation model test of sense of security.

Regression equation (*N* = 1,119)		Fit indicator			Coefficient of significance	
Outcome variable	Predictor variable	$R^{2}$	Adjustment $R^{2}$	F value	β	t
Academic self-efficacy	Constant	0.282	0.281	438.825***	1.755***	24.191
	Social support				0.434***	20.948
Sense of security	Constant	0.025	0.024	28.970***	2.558***	23.460
	Social support				0.168***	5.382
Academic self-efficacy	Constant	0.297	0.295	235.183***	1.513***	17.243
	Social support				0.418***	20.122
	Sense of security				0.094***	4.788

*indicates *p* < 0.05, **indicates *p* < 0.01, ***indicates *p* < 0.001.

**Table 4:** Meditation effects.

Item	Symbol	Meaning	Effect	95% CI		Standard Error SE value	z value/t value	*p* value	Conclusion
				Lower limit	Higher limit				
Social support→sense of security→academic self-efficacy	a*b	Indirect effect	0.016	0.004	0.041	0.010	1.640	0.101	Partial mediation
Social Support→Sense of Security	a	X= > M	0.168	0.107	0.229	0.031	5.382	0.000	
Sense of Security→academic self-efficacy	b	M= > Y	0.094	0.056	0.133	0.020	4.788	0.000	
Social support→academic self-efficacy	c’	Direct effect	0.418	0.377	0.459	0.021	20.122	0.000	
Social support→academic self-efficacy	c	Total effect	0.434	0.393	0.474	0.021	20.948	0.000	

### Moderating effect test

Again, model 59 in the SPSS plug-in macro PROCESS prepared by Hayes in 2017 (72) was used with social support as the independent variable, academic self-efficacy as the dependent variable, sense of security as the mediating variable, and family cultural capital as the moderating variable. The results were shown in Table 5. The analysis of moderating mediating effects involved 2 models, as follows:

**Table 5:** Equation modeling of the role of the regulatory intermediary.

Regression equation (*N* = 1,119)		Fit indicator			Coefficient of significance	
Outcome variable	Predictor variable	$R^{2}$	Adjustment $R^{2}$	F value	β	t
Sense of security	Constant	0.042	0.038	16.134***	3.879***	10.713
	Social support				−0.266*	−2.518
	Family cultural capital				−0.413*	−3.447
	Social support*Family cultural capital				0.134***	4.110
Academic self-efficacy	Constant	0.378	0.375	135.458***	0.529	1.588
	Social support				0.510***	7.622
	Family cultural capital				0.470***	4.485
	Social support*Family cultural capital				−0.070**	−3.383
	Sense of security				0.048	0.745
	Social support*Family cultural capital				0.014	0.735

*indicates *p* < 0.05, **indicates *p* < 0.01, ***indicates *p* < 0.001.

Academic self-efficacy = 0.529 + 0.510*social support +0.470*family cultural capital—0.070*social support*family cultural capital +0.048*sense of security +0.014*sense of security*family cultural capital.

Sense of security = 3.879–0.266* social support - 0.413*family cultural capital +0.134*social support*family cultural capital.

**Figure 3:**
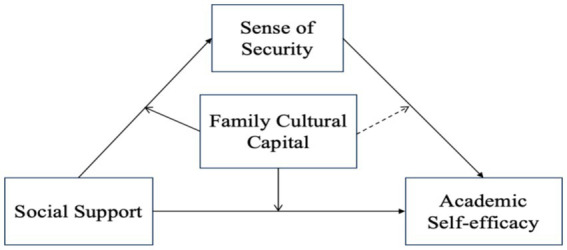
Moderating mediator model.

The results showed that in both models where the dependent variables were sense of security and academic self-efficacy, the interaction terms of social support and family cultural capital showed significance (former: *β* = 0.134, *t* = 4.110, *p* < 0.01; latter: *β* = −0.070, *t* = −3.383, *p* < 0.01), while in the model of academic self-efficacy, sense of security and family cultural self-efficacy interaction terms did not show significance (*β* = 0.014, *t* = 735, *p* > 0.05). This means that among the three pathways of direct and indirect effects, the direct pathway of social support-academic self-efficacy, the indirect pathway of social support-sense of security were subject to moderating effects, and the magnitude of the effect of the moderating variable (cultural capital of the family) differed significantly at different levels, whereas the indirect pathway of sense of security-academic self-efficacy was unaffected by the moderating variable. The modeling diagram of the mediating effects of regulation was shown in Figure 3, with the solid line representing significant regulation and the dashed line representing no regulation.

**Figure 4:**
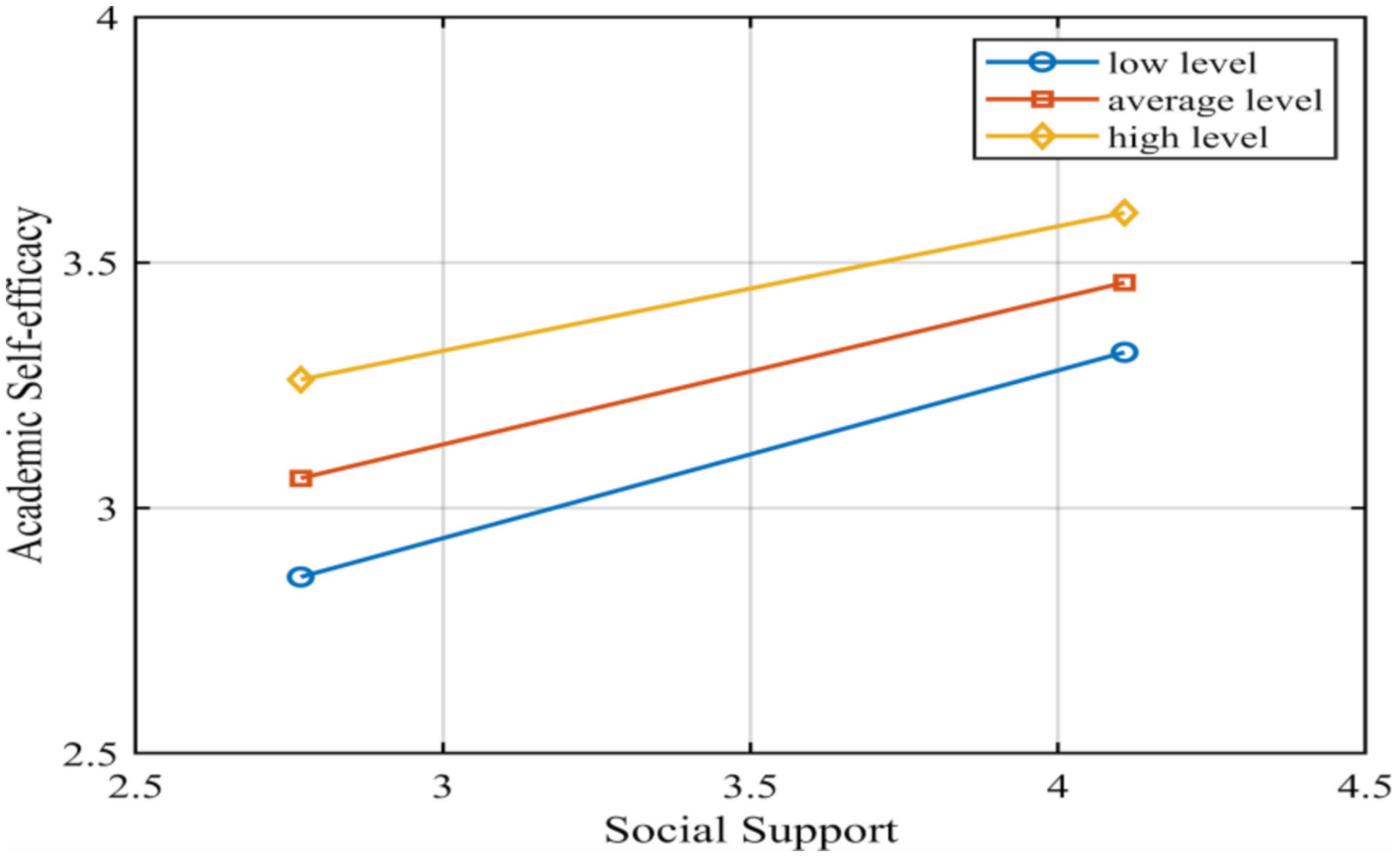
Simple slope plot of conditional direct effects.

In order to test the moderating effect of the moderator variables, a family’s cultural capital score above the mean plus one standard deviation was labeled as a high grouping, and below the mean minus one standard deviation was labeled as a low grouping. Table 6 demonstrated the conditional direct effects model, where conditional direct effects referred to the effect of the independent variable on the dependent variable at different levels of the moderator variable. Table 6 demonstrated the different effect values from low to high levels of family cultural capital, with a gradual and slow decrease in Effect from low to high levels. Further, we could observe a visual representation of this trend of change through the simple slope plot in Figure 4. It could be seen that although the social support-academic self-efficacy impact relationship weakened and the slope became lower as the value of family cultural capital level increased, the level value of academic self-efficacy was significantly higher in the group with high levels of family cultural capital than in the group with low levels. Moreover, the difference in the value of this level showed a more significant performance in comparison with the weak trend of the slope change.

**Table 6:** Conditional direct effects model.

Level	Level value	Effect	SE	*t* value	*p* value	LLCI	ULCI
Low level(-1SD)	2.397	0.342	0.026	13.181	0.000	0.291	0.392
Mean value	3.024	0.298	0.022	13.419	0.000	0.254	0.341
High level(+1SD)	3.651	0.254	0.025	9.952	0.000	0.204	0.304

LLCI refers to the lower limit of the 95% interval of the estimate, ULCI refers to the upper limit of the 95% interval of the estimate.

**Figure 5:**
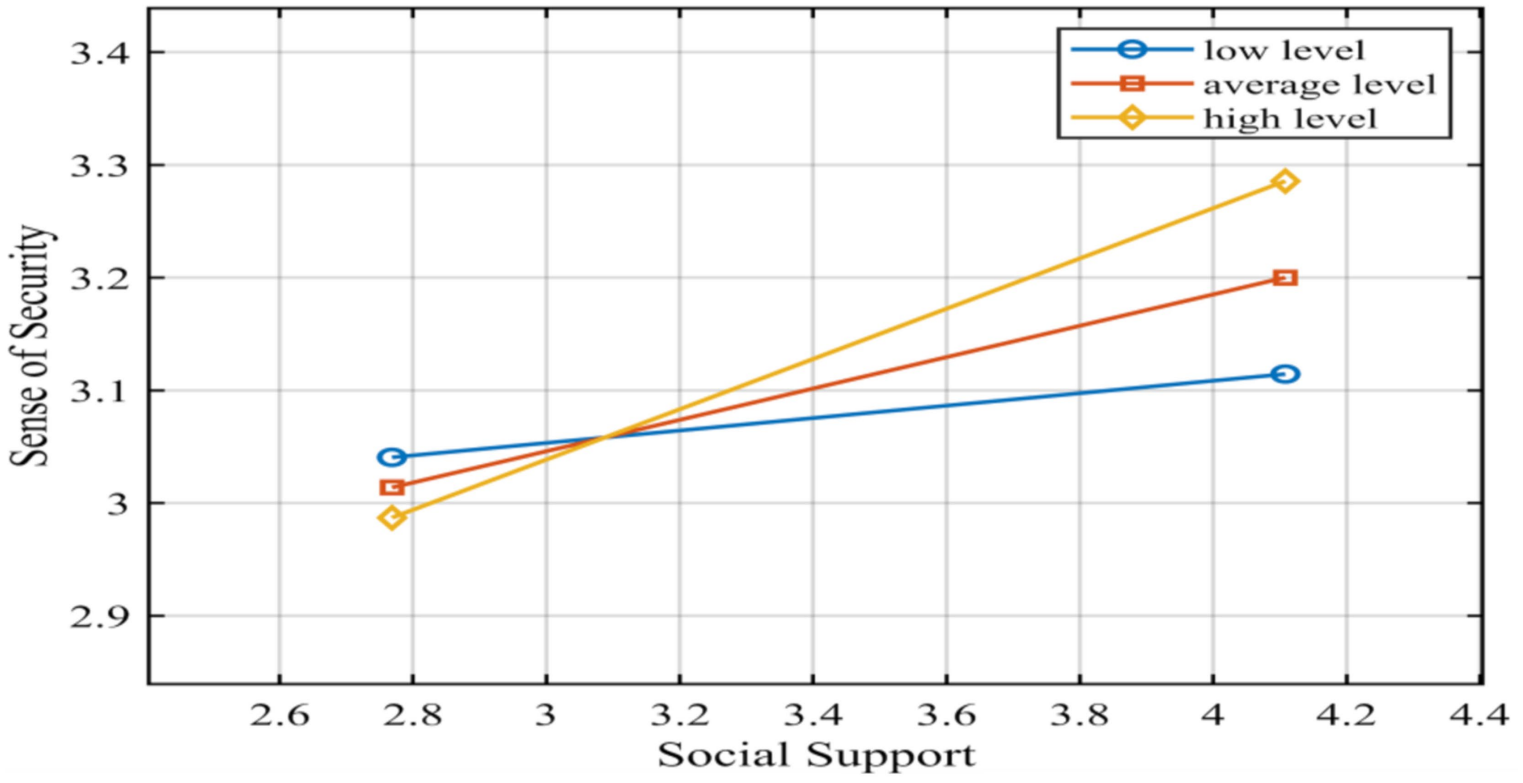
Simple slope plot of conditional indirect effects.

On the other hand, we tested the moderating effect of the indirect path of social support-sense of security. From Figure 5, we could see that the positive predictive relationship between social support and security was stronger in the high family cultural capital subgroups and weaker in the low family cultural capital subgroups; and the academic self-efficacy of the low family cultural capital group was higher than that of the high family cultural capital group in all cases of high social support.

Finally, we performed a moderated mediation analysis for model 59, and as could be seen in Table 7, for the mediating variable of sense of security, its BOOT 95% CI included the number 0 at low levels, implying that there was no mediation at this level; At the mean level, the boot 95% CI did not include the number 0, indicating that there was a mediating effect at this level, and the Effect value was 0.013; at the high level, the boot 95% CI did not include the number 0, implying that there was a mediating effect at this level, and the Effect value was 0.022. In summary, it could be seen that the mediating effect situation was inconsistent, suggesting a moderating mediating effect.

**Table 7:** Conditional indirect effects model.

Mediation variable	Level	Level value	Effect	BootSE	BootLLCI	BootULCI
Sense of security	Low level(-1SD)	2.397	0.005	0.007	−0.005	0.020
	Mean value	3.024	0.013	0.006	0.003	0.026
	High level(+1SD)	3.651	0.022	0.010	0.007	0.044

BootLLCI refers to the lower limit of the 95% interval of Bootstrap sampling, BootULCI refers to the upper limit of the 95% interval of Bootstrap sampling, bootstrap type: percentile bootstrap method.

## Discussion

### The effect of social support on sense of security

The results of this study showed that social support positively predicted sense of security (i.e., college student groups with higher levels of social support would have a higher sense of security). Conversely, college student groups with low social support possessed a lower sense of security. This was similar to the conclusions reached by existing research. People with a high sense of social support tend to have a stable network of psychological support and psychological connections. They can take the initiative to socialize and experience the meaning of common activities with others, increasing their social integration. The sense of belonging that this group of people obtains through the collective is rising, thus contributing to their sense of security (75). Social support is effective in alleviating students’ adverse emotions. At the same time, its role as a stress-buffering factor can effectively reduce the emotional stimulation caused by academic difficulties, thus maintaining students’ psychological resilience. The more social support students receive, the more courageous and confident they are in dealing with difficulties and setbacks encountered in their studies, and the better able they are to overcome the effects of unfavorable factors (76). These students tend to develop a more stable psychological quality and higher psychological self-control. They are more likely to perceive their environment as dependable and trustworthy, thus enhancing their sense of security. On the other hand, students with a lower sense of social support are prone to magnify small personal failures inadvertently. They are more emotionally burdened because of the lack of positive feedback at the social level. Such students are more sensitive to the perceived level of risk in their surroundings, tend to have a sense of powerlessness and loss of control over their studies and life, and have a lower sense of security (30).

A study of international students confirms a similar view. International students with higher levels of social support tend to perceive themselves as students belonging to a social unit. They are more willing to bond and develop important and meaningful relationships with other students within the unit. Their reliance on the social unit for tools, information, and emotional support, among other things, deepens their social relationships, which in turn facilitates the acquisition of a more profound sense of security. For this reason, they perceive their environments to be reliable and stable, to be able to trust, and thus are more willing to interact positively with others across nationalities (77). Higher education workers, parents, and related personnel should provide college students with as much appropriate support as possible so that they can trust and rely on them, which in turn increases their level of security.

### The effect of sense of security on academic self-efficacy

The results of this study showed that sense of security positively predicted academic self-efficacy (i.e., college student groups with higher sense of security had higher levels of academic self-efficacy). Conversely, college student groups with low sense of security had lower academic self-efficacy. Our study drew similar conclusions to existing studies. Sense of security mobilizes students’ interest in pursuing academic opportunities, improves their acceptance of and adaptability to academic challenges, better integrates them into a given academic environment, and ultimately, improves their acquisition of academic self-efficacy in a subtle way (34). The reduced sense of security, on the other hand, makes students hold a considerable degree of mistrust toward their surroundings, which greatly reduces their interest in outdoor and social activities. They tend to believe that they lack the potential and ability to complete their studies, which inadvertently amplifies the interference of negative emotions and their academic self-efficacy is low (36). Another study supports this finding. Under the general environment of the epidemic, college students’ uncertainty about the future increases. The out-of-control social order leads to a sudden decrease in college students’ sense of security, which in turn creates a series of difficulties in their studies and lives. This insecurity affects students’ academic adjustment strategies and triggers more frequent academic burnout. Such students tend to perceive themselves as being unable to deliver a satisfactory performance in accomplishing academic tasks and challenges in this general environment, which reduces their academic self-efficacy (35). Higher education workers, parents and related personnels need to create a safe and comfortable environment (both physical and psychological) for students to grow in, which facilitates their learning and in turn improves their academic self-efficacy.

### The influence of social support on academic self-efficacy

The results of this study showed that social support positively predicted academic self-efficacy. That is, college student groups with higher levels of social support had higher levels of academic self-efficacy and vice versa. A number of studies have confirmed that teacher autonomy support has a significant impact on students’ academic self-efficacy. When students feel autonomy support from their teachers, they experience greater intrinsic motivation and increased engagement in learning, leading to increasing self-adaptation (78). Teachers who provide students with appropriate autonomy support can help students experience the satisfaction of overcoming academic pressures and challenges in the learning process to a greater extent, which in turn enhances students’ self-confidence. Students are more likely to believe that they can rationally use learning strategies to regulate their learning status at different times, and their academic self-efficacy is higher (79). A high level of social support can bring about a more positive emotional experience and a more stable mindset toward learning. A sense of social support fulfills students’ psychological need to help them identify and maintain learning goals in complex learning environments and experience less academic distress. For this reason, students tend to unconsciously amplify their sense of experience and satisfaction in learning, believing that they are able to complete their learning tasks and achieve good grades (37). These students tend to adopt growth-oriented strategies, their personal competencies develop implicitly during the learning process, and they are more willing to believe in their ability to complete academic challenges. Their academic self-efficacy increases (80). While students with insufficient sense of social support are prone to feel the sense of inadequacy brought by the gap between their personal development and their goals in learning, which in turn reduces their motivation and desire to learn, and they do not believe in and are not willing to pay for the appropriate learning behaviors. They tend to adopt negative strategies of avoidance and indifference, and their academic self-efficacy tends to fall into a depressed state easily (81). The study drew similar conclusions to the existing studies. Higher education staff, especially teachers and parents, should provide timely feedback and assistance to students in their professional learning to reduce burnout, helplessness and powerlessness in their learning, so that students can have more successful learning experiences, which in turn will improve their academic self-efficacy.

### Mediating effect of sense of security

The results of this study showed that the sense of security partially mediated mediating role between social support and academic self-efficacy. That is, college student groups with higher social support had a higher ability to feel safe and thus would have a higher degree of academic self-efficacy. It has been established that social support positively predicts sense of security. A sense of social support promotes the development of more adaptive personality traits in students, helping them to enhance the perception of access in environments such as academics or interpersonal relationships. They are more likely to receive positive evaluations of themselves and positive emotional experiences in social situations, and their sense of interpersonal integrity and control is correspondingly higher. They tend to perceive themselves as being in a safer and more controlled atmosphere (82). Students with a high sense of social support will likely to experience perceived commonalities in social interactions (e.g., shared interests). This allows students to embody their emotions and present themselves more harmoniously and also helps to increase trust in each other, resulting in a higher sense of security (83). When faced with stress and difficulties, such students tend to believe that they have adequate adjustment strategies to cope with the challenges. They can deal with issues related to social interactions in a more proactive manner (84).

In addition, a number of studies have confirmed that sense of security positively predicts academic self-efficacy. Students’ sense of insecurity stems from their lack of experience in dealing with the external environment and their perceived lack of adequate capacity as well as resources to address multiple and complex issues. The increase in students’ insecurities during the epidemic has led to an increase in their negative and depressing emotions, which in turn affected their normal learning. They have difficulty regulating their state of self to adapt to the high-intensity academic pace and are less likely to believe that they can accomplish academic challenges, reducing their academic self-efficacy (85). A reduced sense of security affects students’ academic engagement, reduces their academic motivation, and leads to fatigue or futility in learning (52). The less secure students are, the more susceptible they are to negative emotions, and frequent mood drops can significantly affect students’ learning outcomes. In the face of an uncertain future, students find it challenging to engage in learning with enthusiasm and doubt the importance of learning. This may lead to avoidance or refusal to learn and a decrease in their academic self-efficacy (86). Our study confirmed the mediating role of security in this effect of social support on academic self-efficacy.

### Moderating mediating effect of family cultural capital

The results of this study showed that family cultural capital played a moderating role in the mediating effect of social support on academic self-efficacy and moderates the first half of the direct and indirect effects. College student groups with high levels of family cultural capital had a stronger effect of social support on sense of security and significantly higher academic self-efficacy. Families with richer family cultural capital have a higher “propensity” to consume culture in general and culture that is beneficial to academics. These families are richer in classical literature, poetry books, and works of art. These students are able to pass the “cultural discomfort period” more smoothly in their transition to school life. They are more comfortable in their academic life and have a higher sense of well-being (87). Family cultural capital raises students’ educational aspirations. Families with high family cultural capital are more likely to find appropriate extracurricular activities for their students. Parents are also more likely to be involved in activities related to their children’s future academic development. As a result, such students are more likely to accumulate non-cognitive skills such as negotiation, self-confidence, socialization, teamwork, and leadership through cultural activities, and are able to integrate more quickly into new environments with higher and stable levels of personal security (88). These parents have more generous family support and are more likely to encourage their children to study abroad. They encourage their children to try more “challenging” things, and place a higher value on the quality of their study and the experience of living abroad. Children who grow up in this culture are more receptive to the emergence of new things and are also more comfortable with the unexpected challenges of study abroad life and gradually improve their adaptability and their sense of security (89). Family cultural capital positively predicts security.

In addition, students with higher family cultural capital have richer access to cultural resources at the family level. Affordability and accessibility of cultural resources are more readily available, allowing these students to have a more stable academic environment. They are more likely to be academically successful and have a greater sense of academic self-efficacy (90). These parents have a better understanding of how to navigate the education system effectively and are able to convey messages and behaviors to their children in subtle ways that give them an advantage in successfully transitioning to higher education and improving their academic resilience. These students are more likely to achieve better academic results and experience a sense of accomplishment and satisfaction in their studies, which in turn increases their academic self-efficacy (91). At the same time, these parents are relatively well educated and are able to help their children have more resources for their development. Students from families with lower cultural capital, on the other hand, are limited by the cultural capital available to them and their perspectives are more backward. This hinders their academic performance to some extent and their academic self-efficacy is lower (63). Family cultural capital also positively predicts academic self-efficacy.

Our study confirmed that family culture capital played an important moderated role in the direct and indirect effects of social support and academic self-efficacy. Therefore, parents should provide their children with rich family cultural capital as much as possible.

## Contributions, limitations and prospects

### Contributions

College students’ academic self-efficacy is important to their academic development and physical and mental health, and has attracted extensive attention from scholars around the world. This study focuses on the interrelationships and potential mechanisms between social support, sense of security, family cultural capital and academic self-efficacy. The study found that (1) social support significantly increased sense of safety, (2) sense of safety significantly enhanced academic self-efficacy, (3) social support directly contributed to academic self-efficacy, and (4) sense of safety partially mediated the relationship between social support and academic self-efficacy; (5) family cultural capital played a moderating role in the effects of social support on academic self-efficacy, especially in the first half of the direct and indirect effects.

Currently, there are many studies on college students’ academic self-efficacy, and related studies focus on the relationship between academic self-efficacy and academic help-seeking (92, 93), academic procrastination (94, 95), learning strategies and academic achievement (96, 97) and learning burnout (6, 96) and so on. In addition, more studies have explored its mediating role (98–102) and fewer have examined its moderating role (103, 104). There are many studies focusing on the mechanism of social support on college students’ academic self-efficacy. However, in this study, we also explored the mediating and moderating roles of sense of security and family cultural capital in this influence process, which are few in previous studies. Therefore, the above findings in this study are conducive to a more comprehensive and in-depth understanding of the mechanisms influencing college students’ academic self-efficacy among college workers, parents, and related personnel. On the one hand, this study enriches the theoretical research related to social support, college students’ academic self-efficacy, sense of security and family cultural capital to some extent. On the other hand, the conclusions drawn from this study can also provide some practical guidance for college workers, parents and related personnel to enhance college students’ academic self-efficacy.

Firstly, providing more social support for students. In the college learning life, students may encounter various difficulties and challenges. Due to their lack of experience, they need to be surrounded by trustworthy people who can provide timely and appropriate help. They can gain more successful experiences and improve their academic self-efficacy through various forms of social support. Teachers are important others for students, and they have important influence on students’ growth. Therefore, teachers should provide professional support for college students as much as possible, answer students’ questions in a timely manner, inspire and induce students to explore, and learn to discover, analyze and solve problems. At the same time, teachers can also give students more care and support inside and outside the classroom. Colleges should provide more platforms for students to grow and provide them with appropriate teacher support, financial support, equipment support and venue support. Parents, on the other hand, in addition to providing appropriate material support, should also provide students with sufficient moral support, so that students can face the difficulties in the learning process bravely and move forward.

Secondly, enhancing students’ sense of security. The sense of security can affect students’ mental health, which in turn affects their learning status. Colleges should actively build a “campus safety community” to create a favorable campus environment for students’ learning and growth. At the same time, colleges should also create an all-inclusive psychological education model to enhance students’ mental health (105). When students encounter difficulties, they can seek help from relevant school personnel, and will not fall into the negative emotions of helplessness and despair.

No matter how good or bad a student’s performance is and what kind of difficulties he or she is facing, teachers and parents should still look at the student from a developmental perspective, not giving up or getting bored, but being more patient and encouraging, and facing the situation together with the student. Parents should let students know that they are always the most substantial support. As long as students are in need, parents are always around to provide them with timely and selfless care and help. As students’ sense of security is enhanced, they will have more courage and confidence to overcome difficulties.

Thirdly, enriching students’ family cultural capital. This is not only limited to parents providing students with a good material economic foundation, but also emphasizes that they give students spiritual support. A good family environment and cultural atmosphere will influence the healthy growth of students in a subtle way. Parents should set good examples for students and create a good family cultural atmosphere. Where financial conditions permit, parents should purchase good books and study tools at home as much as possible, and develop good habits of reading and studying in their leisure time, thus to set an example for the growth of students. In addition, parents should set reasonable educational expectations, taking into account the characteristics and interests of their students. Parents’ cordial relationship, hard-working attitude toward learning and life and their respect and positive guidance to students can better promote their healthy development.

### Limitations and prospects

This study also has some limitations. First, the limited representativeness of the sample makes it difficult to adequately identify causal relationships between variables because the data are from a cross-sectional survey and convenience sampling methods are used. In addition, in the study, although our samples are from different types of college (including public, private; comprehensive, applied, etc.), we did not analyze the data from different types of colleges in a comparative manner based on the length of the article and time as well as energy constraints. Therefore, future studies should be designed and implemented using a variety of data collection methods. At the same time, future studies should make full use of longitudinal data to further validate the causal relationships of relevant variables to support these findings. In addition, subsequent studies can compare data from different types of colleges to gain a more in-depth and comprehensive understanding of the specific manifestations of this influence mechanism in different colleges, so as to make more targeted recommendations.

## Data Availability

The original contributions presented in the study are included in the article/supplementary material, further inquiries can be directed to the first/corresponding author.
